# Patient-Perceived and Record-Based Concordance of AI-Generated Perioperative Information After Total Hip Arthroplasty: A Cross-Sectional Study with Multidisciplinary Review

**DOI:** 10.3390/medicina62101873

**Published:** 2026-09-27

**Authors:** Mehmet Yağız Yenigün, Ozancan Biçer, Cemre Aydın, Sidar Güneş, Ali Tarık Kutlu, Oktay Adanır

**Affiliations:** Department of Orthopaedics and Traumatology, Bağcılar Training and Research Hospital, University of Health Sciences, Bağcılar 34200, Istanbul, Türkiye

**Keywords:** total hip arthroplasty, artificial intelligence, large language model, patient education, patient experience

## Abstract

*Background and Objectives*: Large language models, a form of artificial intelligence (AI), can answer common patient questions about total hip arthroplasty (THA), but most evaluations emphasize clinician-rated accuracy or patient preference rather than whether generalized recovery statements correspond to patients’ documented postoperative courses. We evaluated a fixed set of ChatGPT 5.1 Thinking responses to 12 investigator-selected questions about elective primary THA from patient, medical-record, and multidisciplinary clinical perspectives, with particular attention to complicated recovery. *Materials and Methods*: In this cross-sectional study with retrospective record review, 101 adults at least 6 months after elective primary THA reviewed a fixed Turkish-language set of ChatGPT 5.1 Thinking answers to 12 investigator-selected questions. Patients rated overall experiential concordance, retrospectively perceived preoperative usefulness, and clarity (0–10). Medical records provided operative duration, first mobilization day, length of stay, and 90-day complications. Three prespecified chart-verifiable timing statements formed a 0–3 timing concordance score. Six multidisciplinary clinicians independently rated all answers during separate 30 min digital assessment sessions. *Results*: Mean overall patient-perceived concordance was 8.44 ± 0.95. Fourteen patients (13.9%) had a complicated recovery and reported lower concordance than those with uncomplicated recovery (7.57 ± 1.22 vs. 8.57 ± 0.83; *p* = 0.0026). The stated ranges for operative duration, early mobilization, and length of stay matched the corresponding records in 93.1%, 94.1%, and 91.1% of patients, respectively; 85.1% met all three timing criteria. Complete three-criterion timing concordance was lower after complicated recovery (28.6% vs. 94.3%; *p* < 0.001). Patient-perceived concordance correlated with the timing concordance score (Spearman ρ = 0.395; *p* < 0.001). Mean multidisciplinary appropriateness across 72 ratings was 8.50 ± 0.50. *Conclusions*: The fixed AI-generated THA information set was rated favorably by patients and clinicians, and its three prespecified timing statements corresponded closely to routine documented recovery. Both experiential and timing concordance were lower after complicated courses. These findings support clinician-reviewed AI information as an adjunct while underscoring that generalized timelines are conditional ranges, not individualized predictions.

## 1. Introduction

Patient education before total hip arthroplasty (THA) is expected to prepare patients for the procedure, common recovery milestones, risks, and the possibility that an individual course may differ from a routine pathway. Artificial intelligence (AI) applications are expanding across orthopedic care [1], and large language models (LLMs) can provide rapid, conversational responses to patient questions. Digital patient-engagement platforms, including chatbots, are also increasingly used to extend perioperative communication and education across the arthroplasty episode of care [2].

Existing THA studies have generally evaluated whether chatbot answers are accurate, appropriate, comprehensive, readable, or preferred by patients. ChatGPT responses to common THA questions have often been judged satisfactory but have required clarification for nuance [3]. In a 60-question comparison, surgeon-rated appropriateness was high for both ChatGPT and arthroplasty-trained nurse responses, although many patients remained uncertain about trusting AI [4]. Other studies have similarly reported useful or generally acceptable AI-generated orthopedic information while identifying incomplete, overly complex, or qualification-sensitive content [5,6,7,8]. Arthroplasty-specific testing has likewise shown that ChatGPT can provide useful support while remaining inferior to adult-reconstruction clinicians on detailed knowledge questions, reinforcing a supplementary rather than substitutive role [9]. Recent work has expanded to physician-supervised informed consent, cross-language evaluation, robot-assisted THA questions, and customized perioperative AI platforms [10,11,12,13].

A separate question is whether generalized AI information resembles what actually happens to patients. This is particularly important in arthroplasty because routine statements such as “1–2 h,” “same-day or next-day mobilization,” or “1–3 days in hospital” may describe a typical pathway while failing to represent patients with medical or surgical complications. Recent arthroplasty data demonstrate clinically relevant differences between preoperative expectations and postoperative experience for pain, mobility, physical therapy, and follow-up [14]. Moreover, even within contemporary THA pathways, the timing of early mobilization is heterogeneous; prospective data suggest that mobilization within the first postoperative day can be appropriate across a range of timings rather than at one deterministic time point [15]. Expectation fulfillment has also been associated with satisfaction after THA [16]. Excluding patients with complicated recovery can therefore make a generalized educational message appear more concordant than it is across the full clinical spectrum.

The purpose of this study was therefore to evaluate a fixed 12-question AI-generated THA information set from three complementary perspectives: patient-perceived concordance with lived recovery, timing concordance with prespecified perioperative events documented in the medical record, and independent multidisciplinary clinical review by reviewers who were not informed of the AI source. A prespecified secondary objective was to determine whether both perceived and timing concordance were lower among patients with complicated postoperative courses. We hypothesized that overall concordance would be high, but that deviations would cluster in complicated recoveries and in timeline-related content identified by the multidisciplinary reviewers.

## 2. Materials and Methods

### 2.1. Study Design and Participants

This was a single-center cross-sectional survey with retrospective record-based comparison. Adults who underwent elective primary THA for primary hip osteoarthritis between December 2024 and December 2025 were identified from institutional surgical records and surveyed during routine postoperative follow-up between April and June 2026. Eligibility was assessed on the individual survey date, and no participant was surveyed before completing 6 months of postoperative follow-up. All questionnaire administration occurred after ethics approval was granted on 15 April 2026. Participants were required to be at least 18 years old, to understand Turkish, and to be able to complete the questionnaire independently. Reporting followed the Strengthening the Reporting of Observational Studies in Epidemiology (STROBE) recommendations for cross-sectional studies [17].

Revision THA and arthroplasty performed for acute trauma or femoral neck fracture were excluded because the educational set was designed for elective primary THA for osteoarthritis. No patient was excluded because of postoperative complications, unplanned readmission, delayed recovery, or reoperation. Of 117 records screened, six did not meet the procedure/indication criteria, 111 patients were eligible, 10 declined participation, and 101 were enrolled and analyzed.

### 2.2. AI-Generated Information Set

Before AI generation, the investigators selected 12 standard topic areas intended to represent the clinical stages and information needs typically encountered during elective primary THA care. The domains were procedure overview, operative duration, anesthesia, hospital stay, early mobilization, pain control, complications, warning symptoms, assistive devices, driving/work, rehabilitation, and long-term precautions/follow-up. Selection was informed by routine clinical encounters and by the question domains used in prior THA chatbot studies [3,4]. The list was fixed before the model was queried; the present study did not independently measure the population frequency of each question.

A standardized Turkish-language answer set was generated once with ChatGPT 5.1 Thinking (OpenAI, San Francisco, CA, USA) through the ChatGPT web interface on 23 March 2026. The model was instructed to answer the 12 investigator-selected questions in concise, patient-friendly Turkish, describe typical perioperative and recovery expectations, include important risks and warning symptoms, identify when professional review was needed, avoid unnecessary technical terminology, and answer each question separately. The exact prompt, all 12 questions, and the complete model answers as presented to participants after spelling, punctuation, and formatting correction are reproduced with English translations in Supplementary Material S1. A single output was used; no answer was regenerated and no clinical statement was added, removed, or substantively rewritten.

Before presentation, only spelling, punctuation, and formatting corrections were permitted. No clinical statement was added or removed after generation. The material was intentionally retained as a fixed stimulus so that patient, chart-based, and professional evaluations referred to the same information.

### 2.3. Patient-Reported Evaluation

After reading all 12 question-answer pairs, participants provided one overall 0–10 score for each of three study-specific items: experiential concordance (“How well does this information match what you actually experienced during your recovery?”), retrospectively perceived preoperative usefulness (“Looking back, how helpful do you think this information would have been before surgery?”), and clarity (“How clear and understandable is this information?”). Higher scores indicated more favorable ratings. These items were developed for this exploratory study, were not psychometrically validated, and did not provide domain-specific assessments of the 12 answers. Participants completed the paper questionnaire without the treating surgeon present and were not informed that the material had been generated by AI before submitting their ratings.

### 2.4. Record-Based Timing Concordance

Electronic records were reviewed for operative duration (skin incision to closure), anesthesia type, first postoperative mobilization day, acute-care length of stay, and postoperative events through 90 days. A complicated recovery was defined as any postoperative complication requiring active treatment, unplanned readmission, or reoperation within 90 days. Examples included transfusion-requiring postoperative anemia, wound complications requiring additional treatment, dislocation requiring closed reduction, symptomatic deep-vein thrombosis, urinary retention requiring recatheterization, and transient acute kidney injury requiring treatment; the observed distribution is reported in Section 3.1. Complication categories were adjudicated from the medical record before linkage to patient questionnaire scores.

Three explicit, chart-verifiable timing statements in the AI material were prespecified for record-based comparison: operative duration of approximately 60–120 min, first mobilization on postoperative day (POD) 0 or 1, and hospital stay of 1–3 days. Each matching criterion contributed one point to a timing concordance score ranging from 0 to 3. This score assessed only these three generalized timing windows and was not a measure of the overall factual accuracy, safety, completeness, or clinical validity of all 12 answers. The complication and warning-sign answers were additionally examined descriptively to determine whether they explicitly represented the categories of adverse events observed in the cohort.

### 2.5. Multidisciplinary Clinical Review

Six clinicians reviewed the same 12 answers individually rather than as a consensus panel: three orthopedic surgeons who performed THA (2, 7, and 15 years of arthroplasty experience), one physiotherapist with 9 years of arthroplasty rehabilitation experience, one orthopedic/perioperative nurse with 12 years of experience, and one anesthesiologist with 10 years of perioperative practice. On 29 April 2026, each reviewer completed an independent 30 min digital assessment in a different room, without discussion or access to another reviewer’s ratings. Reviewers were not informed that the answers had been generated by AI and were also blinded to patient scores, record-based findings, and one another’s ratings.

For each answer, reviewers assigned a 0–10 clinical appropriateness score, answered a prespecified yes/no item asking whether any wording could be potentially misleading or unsafe if used without clinician qualification, and provided free-text comments. “Flagged” denotes a yes response to this prespecified item. The reviewers were not asked to reach consensus before or after scoring.

Free-text comments were organized by the 12 question domains and summarized descriptively. One study investigator responsible for data analysis grouped comments expressing similar recommendations and drafted the summaries. A second study investigator verified the groupings, summary wording, and reported reviewer counts against the original written comments. Differences in interpretation were resolved by returning to the source comments; differing views and isolated safety concerns were retained. Theme frequencies are reported only for selected quantified themes. For each reported theme count, each reviewer was counted at most once, and only an explicitly expressed written recommendation or view contributed to the count. Silence and numerical appropriateness scores were not treated as endorsement. The denominator for reported theme counts was six reviewers. This descriptive synthesis did not require the reviewers to reach consensus or change their original ratings.

“Main recurrent reviewer theme” is a descriptive summary of written comments across all 12 domains. Where shown, theme counts such as 6/6 indicate the number of reviewers explicitly expressing the summarized recommendation or view. An omitted count means that a frequency is not reported; it implies neither zero endorsements nor unanimity. These counts are separate from the yes/no flag counts: a reviewer could recommend clearer wording without flagging the answer as potentially misleading or unsafe. Supplementary Table S3 provides expanded paraphrased summaries for six selected domains, with counts for selected additional themes; it is not an exhaustive list or a collection of verbatim quotations.

### 2.6. Statistical Analysis

Continuous data are summarized as mean ± standard deviation (SD) and median with interquartile range (IQR) where appropriate; categorical variables are presented as counts and percentages. Mean patient scores are reported with two-sided 95% confidence intervals. Wilson 95% confidence intervals were calculated for timing concordance proportions.

Patient scores and the 0–3 timing concordance score were compared between uncomplicated and complicated recovery groups using two-sided Mann–Whitney U tests; rank-biserial correlation was used as the effect size for the primary patient-score comparison. The association between patient-perceived concordance and the timing concordance score was assessed with Spearman correlation. Categorical concordance proportions were compared using Fisher exact tests. The recovery-group comparison was prespecified to examine how well generalized timing windows represented routine versus complicated postoperative trajectories; it was not intended to estimate a causal effect of complications on concordance.

Reviewer scores were summarized overall and by question. The overall multidisciplinary mean and SD were calculated across all 72 individual appropriateness ratings (12 answers × 6 reviewers), using the arithmetic mean and sample SD (denominator *n* − 1). Answer-level means were calculated across the six reviewers. Inter-rater reliability was estimated with a two-way random-effects, absolute-agreement intraclass correlation coefficient for a single reviewer (ICC(A,1)) and the mean of six reviewers (ICC(A,6)) [18]. Because only 12 answer targets were rated, 95% confidence intervals were estimated by bootstrap resampling of answer targets. Two-sided *p* < 0.05 was considered statistically significant. Secondary analyses were exploratory, and p values were not adjusted for multiplicity. Analyses were performed using Python 3.13 with NumPy, SciPy 1.17.0, and statsmodels. The available cohort defined the analysis set; no a priori power calculation was performed.

### 2.7. Ethics

The study was approved by the institutional Clinical Research Ethics Committee (approval no. 2026/04/15/104; 15 April 2026). All study procedures involving participants were conducted after approval. Participants were not informed that the perioperative educational content was AI-generated before completing the questionnaire. All participants provided written informed consent before questionnaire administration.

## 3. Results

### 3.1. Participants and Postoperative Complexity

All 101 enrolled participants completed the three patient-reported ratings, and no data were missing for the three prespecified timing concordance variables. Mean age was 65.45 ± 7.68 years (median 65, IQR 61–72; range 44–82), and 52 participants (51.5%) were women. Fourteen participants (13.9%) met the definition of complicated recovery. The complication profile comprised transfusion-requiring postoperative anemia (*n* = 4), wound complications requiring additional treatment (*n* = 3), dislocation requiring closed reduction (*n* = 2), symptomatic deep-vein thrombosis (*n* = 2), urinary retention requiring recatheterization (*n* = 2), and transient acute kidney injury requiring treatment (*n* = 1). Four patients had an unplanned readmission and one underwent reoperation for a wound complication (Table 1).

### 3.2. Patient-Perceived Concordance

Overall experiential concordance was 8.44 ± 0.95 (95% CI 8.25–8.62; median 9, IQR 8–9), retrospectively perceived preoperative usefulness was 8.95 ± 0.77 (95% CI 8.80–9.10; median 9, IQR 8–9), and clarity was 8.48 ± 0.98 (95% CI 8.28–8.67; median 8, IQR 8–9) (Table 2).

Patients with uncomplicated recovery reported higher experiential concordance than patients with complicated recovery (8.57 ± 0.83 vs. 7.57 ± 1.22; Mann–Whitney U = 897, *p* = 0.0026; rank-biserial r = 0.473). In contrast, retrospectively perceived preoperative usefulness (8.97 ± 0.75 vs. 8.86 ± 0.86; *p* = 0.564) and clarity (8.53 ± 1.01 vs. 8.14 ± 0.66; *p* = 0.141) did not differ significantly between recovery groups.

### 3.3. Record-Based Timing Concordance

Mean operative duration was 92.99 ± 17.89 min; 94 of 101 patients (93.1%; 95% CI 86.4–96.6%) fell within the AI-stated 60–120 min range. First mobilization occurred on POD 0 in 39 patients and POD 1 in 56; 95 of 101 (94.1%; 95% CI 87.6–97.2%) therefore matched the early-mobilization statement. Median length of stay was 2 days (IQR 2–3), and 92 of 101 patients (91.1%; 95% CI 83.9–95.2%) stayed 1–3 days (Table 3). These proportions describe correspondence only for the three prespecified timing statements.

Eighty-six patients (85.1%; 95% CI 76.9–90.8%) met all three timing criteria. Complete three-criterion timing concordance was observed in 82 of 87 uncomplicated recoveries (94.3%) but only 4 of 14 complicated recoveries (28.6%; Fisher exact *p* < 0.001). The mean timing concordance score was 2.78 ± 0.61 overall, 2.92 ± 0.38 after uncomplicated recovery, and 1.93 ± 1.00 after complicated recovery (*p* < 0.001). Patient-perceived concordance correlated with the timing concordance score (Spearman ρ = 0.395; *p* < 0.001).

The complication and warning-sign answers explicitly represented the event category documented in 11 of the 14 patients with complications (78.6%). The omitted categories were urinary retention (*n* = 2) and transient acute kidney injury (*n* = 1). All adverse-event categories that led to unplanned readmission or reoperation were represented in the AI-generated risk or warning-sign content.

### 3.4. Multidisciplinary Review

The six reviewers contributed 72 appropriateness ratings across 12 answers. The overall arithmetic mean across the 72 individual ratings was 8.50, with a sample SD of 0.50. Answer-level means ranged from 7.83 for hospital stay to 9.25 for pain control. ICC(A,1) was 0.663 (bootstrap 95% CI 0.395–0.796), and ICC(A,6) was 0.922 (bootstrap 95% CI 0.777–0.954) (Table 4).

The most frequent flags concerned timeline wording: three reviewers flagged operative duration, three flagged hospital stay, and two flagged early mobilization. Free-text comments recommended more prominent qualification of numerical timelines and supported individualized decisions regarding assistive-device discontinuation and return to driving or work.

## 4. Discussion

This study triangulated overall patient experience, three chart-verifiable perioperative timing events, and multidisciplinary review of a fixed AI-generated information set. The set received favorable patient and clinician ratings, while the three timing statements corresponded more closely to routine than to complicated postoperative courses. This finding does not constitute comprehensive record-based assessment of all 12 answers. Rather, it identifies clinically important boundary conditions for generalized timelines. Independent reviewer comments converged on the same timing domains and supported more prominent qualification of numerical ranges.

Prior studies have established that LLMs can provide many useful or acceptable responses relevant to arthroplasty patient education, but they also consistently demonstrate a need for clarification, limitations in completeness, or uncertainty regarding trust [3,4,5,6,7,8]. In Arthroplasty Today, ChatGPT 4 achieved 66.9% accuracy on 299 arthroplasty self-assessment questions, compared with 84.3% for an adult-reconstruction fellow and 85.3% for an attending surgeon, supporting use as an adjunct rather than a substitute for specialist judgment [9]. Turkish-language THA discharge material generated with AI has also shown favorable expert assessments and readability [8]. More recent studies have evaluated physician-supervised AI for informed consent and customized AI platforms for perioperative arthroplasty questions [10,13]. Digital engagement platforms that include chatbots may broaden perioperative communication, but their value depends on how well generic content is matched to an individual clinical trajectory [2]. The present design extends that literature by asking whether generalized educational claims align with actual patient-level perioperative events rather than with expert judgment alone.

Recent reports in Medicina provide a useful parallel to these findings. In hip arthroscopy, ChatGPT-3.5 responses to standardized patient questions received high expert ratings for relevance, accuracy, and clarity, but the authors still emphasized limitations in postoperative detail and variability [19]. In hand surgery, ChatGPT-4o-generated patient information was rated favorably by both patients and physicians, while gaps in completeness and individualized nuance remained evident [20]. A patient-and-public-involvement study of hip and knee replacement rehabilitation further underscored the importance of incorporating patient priorities when designing recovery information and interventions [21]. Beyond orthopedics, comparative evaluation of large language models for intraoperative decision support has likewise demonstrated that model performance is context-dependent and should remain subject to professional oversight [22]. Together, these observations support the distinction between high apparent informational quality and clinically individualized validity.

The distinction between routine and complicated recovery is clinically important. General statements about a 60–120 min operation, POD 0–1 mobilization, and a 1–3-day stay were consistent with most observed courses, yet complete timing concordance fell from 94.3% in uncomplicated recovery to 28.6% in complicated recovery. This comparison should be interpreted as a boundary test of generalized timelines rather than as evidence that complications independently cause discordance. The hospital-stay answer itself was explicitly conditional on an uncomplicated course; therefore, lower concordance in the complicated group is partly structural rather than an unexpected model failure. Postoperative complications can prolong hospitalization or delay mobilization, while perioperative complexity may also affect operative duration. Clinically, this expected departure illustrates why generic timetables require prominent qualification. This interpretation is consistent with evidence that preoperative expectations may diverge from postoperative experience across pain, mobility, rehabilitation, and follow-up domains [14] and with prospective THA data showing heterogeneity in early mobilization timing [15].

The patient-reported findings provide complementary but exploratory evidence. Experiential concordance was approximately one point lower in the complicated group, whereas retrospectively perceived preoperative usefulness and clarity remained high. The latter endpoint is a hindsight-based judgment obtained at least 6 months after surgery, not a prospective demonstration that the material improved preoperative knowledge, decision-making, anxiety, adherence, or recovery. Moreover, because only three overall study-specific items were administered, the data cannot identify which of the 12 domains drove favorable or unfavorable ratings.

The independent multidisciplinary review provided a complementary clinical assessment. Previous THA evaluations have frequently relied on surgeons alone [3,4], whereas reviewers from surgery, anesthesia, nursing, and rehabilitation can examine different components of perioperative care. The principal concern was not complete absence of conditional wording; it was that numerical ranges could remain more salient than their qualifiers. Blinding reviewers to the AI source may have reduced the influence of pre-existing favorable or unfavorable attitudes toward generative AI.

These findings support a clinician-reviewed template model rather than autonomous patient counseling. A practical workflow would use an LLM to generate concise baseline information, followed by local clinical review to foreground and standardize qualifiers such as “for many patients,” “if medically stable,” and “depending on your surgeon’s protocol and your recovery.” Complication and red-flag content should be deliberately reviewed for omissions. For patients with atypical trajectories, general educational material should be supplemented with individualized instructions rather than presented as a prediction.

This study has limitations. It was a single-center study of one static output from one model at one time. The 12 standard question domains were selected by the investigators to represent commonly encountered clinical stages, but their population frequency was not measured and the set was not developed through formal patient involvement or external consensus. The patient-reported outcomes were study-specific and not psychometrically validated; no domain-specific ratings were obtained, and postoperative responses are susceptible to recall and hindsight bias. Retrospectively perceived preoperative usefulness is hypothetical and should not be interpreted as demonstrated educational effectiveness. There was no surgeon-authored, institution-authored, or conventional patient-education comparator, so comparative effectiveness, superiority, or noninferiority cannot be inferred. Blinding patients and reviewers to the AI source may have reduced source-related expectation bias but did not permit evaluation of acceptability or trust when AI authorship is disclosed. The complicated subgroup was small, making subgroup estimates imprecise. Because complications may themselves prolong mobilization or hospitalization, group differences should be interpreted as a stress test of generalized timing statements rather than as an independent prognostic association. The record-based timing concordance score covered only three explicit statements and does not establish comprehensive factual validity of all 12 answers. Complication-category coverage was descriptive, not an assessment of risk calibration. Health literacy, education, digital literacy, comorbidity burden, and socioeconomic factors were not measured. Finally, the ICC depends on the specific 12 answers and reviewer mix. The study was not designed to evaluate sex- or gender-based differences in concordance, so differential performance across these groups should be examined in larger cohorts.

## 5. Conclusions

A fixed AI-generated answer set addressing 12 preselected THA questions received favorable overall ratings from patients and multidisciplinary clinicians. Three prespecified timing statements corresponded closely to routine documented recovery but less closely to complicated courses. Because record-based comparison covered only operative duration, early mobilization, and hospital stay, the findings should not be interpreted as record-based assessment of all content domains. The results support clinician-reviewed AI information as an adjunct to THA education and support presenting timelines as conditional ranges rather than individualized predictions.

## Figures and Tables

**Table 1:** Participant characteristics and postoperative complexity.

Characteristic	Value
Participants analyzed	101
Age, years	65.45 ± 7.68; median 65 (IQR 61–72); range 44–82
Women	52 (51.5%)
Men	49 (48.5%)
Uncomplicated recovery	87 (86.1%)
Complicated recovery	14 (13.9%)
Unplanned readmission within 90 days	4 (4.0%)
Reoperation within 90 days	1 (1.0%)
Operative duration, min	92.99 ± 17.89
First mobilization, POD 0/POD 1/POD ≥ 2	39/56/6
Length of stay, days	median 2 (IQR 2–3); range 1–7

Note: Complicated recovery was defined as any postoperative complication requiring active treatment, unplanned readmission, or reoperation within 90 days. IQR, interquartile range; POD, postoperative day.

**Table 2:** Patient-reported ratings overall and by postoperative recovery group.

Outcome (0–10)	Overall (*n* = 101)	Uncomplicated (*n* = 87)	Complicated (*n* = 14)	*p* Value
Experiential concordance	8.44 ± 0.95	8.57 ± 0.83	7.57 ± 1.22	0.0026
Retrospectively perceived preoperative usefulness	8.95 ± 0.77	8.97 ± 0.75	8.86 ± 0.86	0.564
Clarity	8.48 ± 0.98	8.53 ± 1.01	8.14 ± 0.66	0.141

Note: Values are mean ± SD. Two-sided Mann–Whitney U tests compare recovery groups. For experiential concordance, U = 897 and rank-biserial r = 0.473 (uncomplicated > complicated). SD, standard deviation; *n*, number of participants.

**Table 3:** Record-based timing concordance with prespecified AI-generated recovery windows.

Criterion	Overall	Uncomplicated	Complicated	*p* Value
Operative duration 60–120 min	94/101 (93.1%; 86.4–96.6)	84/87 (96.6%)	10/14 (71.4%)	0.0066
Mobilization POD 0–1	95/101 (94.1%; 87.6–97.2)	86/87 (98.9%)	9/14 (64.3%)	<0.001
Length of stay 1–3 days	92/101 (91.1%; 83.9–95.2)	84/87 (96.6%)	8/14 (57.1%)	<0.001
All three criteria	86/101 (85.1%; 76.9–90.8)	82/87 (94.3%)	4/14 (28.6%)	<0.001
Timing concordance score, 0–3	2.78 ± 0.61	2.92 ± 0.38	1.93 ± 1.00	<0.001

Note: Parentheses for overall binary criteria show percentage and Wilson 95% CI. Fisher exact tests compare binary proportions; the Mann–Whitney U test compares the 0–3 score. AI, artificial intelligence; CI, confidence interval; POD, postoperative day.

**Table 4:** Multidisciplinary appropriateness ratings and reviewer flags/comments.

Answer Domain	Mean Score	Flagged Reviewers	Main Recurrent Reviewer Theme
Procedure overview	8.25	0/6	Approach and implant choice should be individualized
Operative duration	8.08	3/6	6/6: make existing operative-time qualifier more explicit
Anesthesia	8.75	0/6	Patient-specific contraindications should be acknowledged
Hospital stay	7.83	3/6	6/6: foreground existing medical/functional discharge qualifiers
Early mobilization	8.33	2/6	6/6: make medical/surgical causes of delay explicit
Pain control	9.25	0/6	Medication contraindications should be individualized
Complications	8.75	0/6	Quantitative risk context suggested
Warning symptoms	9.08	0/6	No major safety concern
Assistive devices	8.33	1/6	Retain function-based, non-fixed discontinuation criteria
Driving/return to work	8.00	2/6	Separate driving and work contingencies more explicitly
Rehabilitation	8.75	0/6	Program should be individualized
Long-term precautions/follow-up	8.58	1/6	Weight management and follow-up should be explicit

Note: Six reviewers rated each answer on a 0–10 appropriateness scale. “Flagged reviewers” denotes reviewers answering yes to the prespecified potentially misleading/unsafe wording item. The overall mean and sample SD were calculated across all 72 individual ratings: 8.50 ± 0.50. ICC(A,1) = 0.663 (bootstrap 95% CI 0.395–0.796); ICC(A,6) = 0.922 (bootstrap 95% CI 0.777–0.954). The final column summarizes written comments; counts such as 6/6 refer to reviewers explicitly expressing the stated recommendation or view. These theme counts are distinct from the yes/no flag counts. Theme frequencies are reported selectively in Table 4 and Supplementary Table S3; an omitted count implies neither zero endorsements nor unanimity. SD, standard deviation; ICC, intraclass correlation coefficient.

## Data Availability

The de-identified data supporting the findings of this study are available from the corresponding author on reasonable request, subject to applicable ethical and institutional requirements.

## Supplementary Materials

The following supporting information can be downloaded at https://www.mdpi.com/article/10.3390/medicina62101873/s1, Supplementary Material S1 contains the generation prompt, the complete 12-question Turkish-language information set with English translations, multidisciplinary reviewer ratings, and selected paraphrased summaries of reviewer comments. The supplementary file contains Table S1, “Twelve-question Turkish information set and English translation”; Table S2, “Multidisciplinary reviewer ratings”; and Table S3, “Selected descriptive summaries of reviewer comments and recommended clarifications”. The question domains were informed by references [3,4].
