# Supplementary material for: Patient-Perceived and Record-Based Concordance of AI-Generated Perioperative Information After Total Hip Arthroplasty: A Cross-Sectional Study with Multidisciplinary Review
Source: Medicina (Kaunas). 2026 Sep 27;62(10):1873. doi: 10.3390/medicina62101873 (PMC13706683; doi:10.3390/medicina62101873)
Supplement: Supplementary file 1 [file medicina-62-01873-s001.zip › medicina-4536571-supplementary.pdf]

Supplementary Material S1

Twelve-question AI-generated perioperative information set and multidisciplinary reviewer ratings

Accompanies: "Patient-Perceived and Record-Based Concordance of AI-Generated Perioperative Information After Total Hip Arthroplasty: A Cross-Sectional Study with Multidisciplinary Review"

Generation prompt

Generation procedure and question selection

Before AI generation, the investigators selected 12 standard topic areas intended to represent commonly encountered clinical stages and information needs during elective primary THA care. Selection was informed by routine clinical encounters and prior THA chatbot question domains (refs. 3 and 4 of the main manuscript). The list was fixed before generation. ChatGPT 5.1 Thinking was queried once through the ChatGPT web interface on 23 March 2026. A single output was used; no answer was regenerated. The responses reproduced below are the versions presented to participants after spelling, punctuation, and formatting correction; no clinical statement was added, removed, or substantively rewritten.

> *"Please answer the following 12 common questions from a patient undergoing elective primary total hip arthroplasty for hip osteoarthritis. Answer in Turkish using concise, patient-friendly language. Explain typical perioperative and recovery expectations, important risks and warning symptoms, and when professional review is needed. Avoid unnecessary technical terminology. Answer each question separately."*

The 12 Turkish questions listed in Table S1 were appended to this prompt in the order shown. Table S1 contains the fixed, minimally copy-edited responses presented to every participant and reviewer.

Table S1. Twelve-question Turkish information set and English translation

Domain	Turkish question	Turkish answer	English question	English answer
1. Procedure overview	Total kalça protezi ameliyatı nasıl yapılır?	Kalçada aşınmış eklem yüzeyleri çıkarılır ve yerlerine yapay eklem parçaları yerleştirilir. Genellikle kalça yuvasına bir kap, uyluk kemiğine bir sap ve bunların arasında hareket eden bir baş yerleştirilir. Amaç ağrıyı azaltmak, hareketi artırmak ve günlük yaşamı kolaylaştırmaktır. Cerrahi yaklaşım ve kullanılan protez tipi hastaya göre değişebilir.	How is total hip replacement performed?	The worn joint surfaces are removed and replaced with artificial joint components. A cup is usually placed in the socket, a stem in the femur, and a head connects the components. The goal is to reduce pain, improve movement, and make daily activities easier. The surgical approach and implant type may vary by patient.
2. Operative duration	Ameliyat ne kadar sürer?	Primer kalça protezi ameliyatı çoğu hastada yaklaşık 1–2 saat sürer. Süre hastanın anatomisine, cerrahi tekniğe ve ameliyat sırasında karşılaşılan güçlüklerle birlikte uzayabilir.	How long does the operation take?	Primary total hip arthroplasty takes approximately 1–2 hours in most patients. The duration can be longer depending on anatomy, surgical technique, and difficulties encountered during surgery.

Domain	Turkish question	Turkish answer	English question	English answer
3. Anesthesia	Hangi anestezi uygulanır?	Ameliyat genel anestezi veya belden uyuşturma olarak bilinen spinal/bölgesel anestezi ile yapılabilir. Seçim; sağlık durumunuz, kullandığınız ilaçlar, anestezi ekibinin değerlendirmesi ve cerrahi planınıza göre yapılır.	What type of anesthesia is used?	The operation can be performed under general anesthesia or spinal/regional anesthesia. The choice depends on your health, medications, anesthesiology assessment, and surgical plan.
4. Hospital stay	Hastanede kaç gün kalırım?	Sorunsuz ilerleyen primer kalça protezi ameliyatlarından sonra çoğu hasta yaklaşık 1–3 gün içinde taburcu edilir. Ağrı kontrolü, yürüme güvenliği, tıbbi durum ve evde destek gereksinimi taburculuk zamanını değiştirebilir.	How long will I stay in the hospital?	After an uncomplicated primary hip replacement, many patients are discharged within about 1–3 days. Pain control, walking safety, medical condition, and support at home can change discharge timing.
5. Early mobilization	Ne zaman ayağa kalkar ve yürürüm?	Çoğu hastada ameliyat günü veya ertesi gün yürüteç ya da koltuk değneği ile ayağa kalkma hedeflenir. İlk amaç güvenli şekilde yataktan kalkmak, kısa mesafe yürümek ve uygun hastalarda merdiven eğitimine başlamaktır. Yük verme durumu ve hareket kısıtlamaları cerrahınızın planına göre değişebilir.	When will I stand and walk?	Most patients are encouraged to stand and begin walking with a walker or crutches on the day of surgery or the next day. The first goals are safe transfers, short-distance walking, and stair training when appropriate. Weight-bearing and movement restrictions may vary according to the surgical plan.
6. Pain control	Ameliyat sonrası ağrı nasıl kontrol edilir?	İlk günlerde ağrı beklenir ve genellikle zamanla azalır. Ağrı kontrolünde parasetamol, uygun hastalarda antiinflamatuar ilaçlar, gerektiğinde daha güçlü ağrı kesiciler ve bazı hastalarda bölgesel anestezi yöntemleri birlikte kullanılabilir. Amaç ağrıyı tamamen sıfırlamak değil, dinlenme ve hareketi mümkün kılacak düzeye indirmektir.	How is pain controlled after surgery?	Pain is expected during the first days and usually decreases over time. Treatment may combine acetaminophen, anti-inflammatory medication when appropriate, stronger analgesics when needed, and regional techniques in selected patients. The goal is not necessarily zero pain, but pain control that allows rest and movement.
7. Complications	Başlıca riskler ve komplikasyonlar nelerdir?	Önemli riskler arasında enfeksiyon, kanama veya kansızlık, bacak ya da akciğerde pıhtı, kalçanın çıkması, protez çevresi kırık, yara iyileşme sorunları, sinir-damar yaralanması ve uzun dönemde gevşeme veya aşınma bulunur. Anesteziye ve diğer hastalıklarınıza bağlı tıbbi sorunlar da gelişebilir. Kişisel risk yaş, kilo, diyabet, sigara kullanımı ve diğer sağlık sorunlarına göre değişir.	What are the main risks and complications?	Important risks include infection, bleeding or anemia, blood clots in the leg or lungs, dislocation, periprosthetic fracture, wound-healing problems, nerve or vessel injury, and long-term loosening or wear. Medical problems related to anesthesia or other health conditions may also occur. Individual risk varies with age, weight, diabetes, smoking, and other health conditions.
8. Warning symptoms	Taburculuk sonrası hangi belirtilerde doktora başvurmalıyım?	Ateş, giderek artan yara kızarıklığı veya akıntı, ani veya belirgin şekilde artan kalça ağrısı, bacakta yeni şişlik, göğüs ağrısı, nefes darlığı, düşme sonrası şekil bozukluğu ya da yürüyememe gibi durumlarda sağlık ekibiyle hızlı şekilde iletişime geçilmelidir. Ciddi nefes darlığı veya göğüs ağrısı acil değerlendirme gerektirir.	Which symptoms should prompt medical review after discharge?	Contact the care team promptly for fever, increasing wound redness or drainage, sudden or markedly worsening hip pain, new leg swelling, chest pain, shortness of breath, deformity after a fall, or inability to walk. Severe shortness of breath or chest pain requires urgent evaluation.

Domain	Turkish question	Turkish answer	English question	English answer
9. Assistive devices	Yürüteç veya bastonu ne kadar kullanırım?	Yürüteç, koltuk değneği veya bastonun ne kadar süre kullanılacağı kişiden kişiye değişir. Birçok hasta ilk haftalarda daha bağımsız yürümeye ilerler; yardımcı cihazı bırakma kararı denge, kas gücü, ağrı, topallama ve yürüme güvenliğine göre verilmelidir.	How long will I need a walker or cane?	The duration of walker, crutch, or cane use varies between patients. Many people progress toward more independent walking over the first several weeks; discontinuation should depend on balance, strength, pain, limping, and walking safety.
10. Driving / work	Ne zaman araba kullanabilir veya işe dönebilirim?	Araba kullanma ve işe dönüş için tek bir süre yoktur. Ameliyat edilen taraf, ağrı kesici kullanımı, güvenli fren yapabilme, yürüme durumu ve işinizin fiziksel gereksinimleri önemlidir. Masa başı işe dönüş genellikle fiziksel işlerden daha erken olabilir. Cerrahınızın kontrolünde onay almak en güvenli yaklaşımdır.	When can I drive or return to work?	There is no single timetable for driving or returning to work. The operated side, pain medication use, ability to brake safely, walking status, and physical demands of the job are important. Return to desk work is often earlier than return to physical work. Confirmation at follow-up is the safest approach.
11. Rehabilitation	Fizik tedavi ve egzersiz nasıl ilerler?	Rehabilitasyon genellikle güvenli yürüme, kalça çevresi kaslarını güçlendirme, denge ve günlük yaşam aktivitelerine dönüş üzerine kurulur. Egzersizlerin yoğunluğu ve ilerleme hızı ağrı, kas gücü, cerrahi yaklaşım ve tıbbi duruma göre ayarlanır. Cerrah ve fizyoterapistin önerileri önceliklidir.	How does physical therapy and exercise progress?	Rehabilitation usually focuses on safe walking, strengthening muscles around the hip, balance, and return to daily activities. Exercise intensity and progression are adjusted according to pain, strength, surgical approach, and medical condition. Guidance from the surgeon and physiotherapist takes priority.
12. Long-term precautions / follow-up	Uzun vadede nelere dikkat etmeliyim?	Yürüyüş ve yüzme gibi düşük etkili aktiviteler çoğu hasta için uygundur. Bazı hareket kısıtlamaları cerrahi yaklaşım ve kalçanın stabilitesine göre değişebilir. Kilo kontrolü, düşmeden korunma, yüksek darbeli aktiviteler konusunda dikkatli olma ve yeni ağrı, topallama veya fonksiyon kaybında ortopedi kontrolüne başvurma önemlidir. Düzenli takip protezin durumunun değerlendirilmesine yardımcı olur.	What should I consider in the long term?	Low-impact activities such as walking and swimming are suitable for many patients. Some movement restrictions vary with surgical approach and hip stability. Weight management, fall prevention, caution with high-impact activities, and orthopedic review for new pain, limping, or functional loss are important. Periodic follow-up helps assess the implant.

Table S2. Multidisciplinary reviewer ratings

Reviewer procedure: On 29 April 2026, the three orthopaedic surgeons, physiotherapist, orthopaedic/perioperative nurse, and anesthesiologist each completed an independent 30-minute digital rating session in a different room. Reviewers were not informed that the responses were AI-generated and had no access to patient ratings, record-based findings, or one another's scores.

Domain	Ortho 1	Ortho 2	Ortho 3	Physio	Nurse	Anesthesia	Mean	Flags
Procedure overview	8.0	8.5	8.0	8.5	8.0	8.5	8.25	0/6
Operative duration	7.5	8.0	8.0	8.5	8.0	8.5	8.08	3/6

Domain	Ortho 1	Ortho 2	Ortho 3	Physio	Nurse	Anesthesia	Mean	Flags
Anesthesia	8.5	9.0	8.5	9.0	8.5	9.0	8.75	0/6
Hospital stay	7.5	8.0	7.5	8.0	8.0	8.0	7.83	3/6
Early mobilization	8.0	8.5	8.0	9.0	8.5	8.0	8.33	2/6
Pain control	9.0	9.5	9.0	9.0	9.5	9.5	9.25	0/6
Complications	8.5	9.0	8.5	8.5	9.0	9.0	8.75	0/6
Warning symptoms	9.0	9.0	9.0	9.0	9.5	9.0	9.08	0/6
Assistive devices	8.0	8.5	8.0	9.0	8.5	8.0	8.33	1/6
Driving / return to work	7.5	8.0	8.0	8.5	8.0	8.0	8.00	2/6
Rehabilitation	8.5	9.0	8.5	9.0	9.0	8.5	8.75	0/6
Long-term precautions / follow-up	8.5	8.5	8.5	9.0	8.5	8.5	8.58	1/6

Note: Ortho, orthopaedic surgeon; Physio, physiotherapist; Flags, number of reviewers flagging potentially misleading or unsafe wording; ICC, intraclass correlation coefficient; CI, confidence interval.

Reviewer summary: overall mean across 72 ratings = 8.50 ± 0.50; ICC(A,1) = 0.663 (bootstrap 95% CI 0.395–0.796); ICC(A,6) = 0.922 (bootstrap 95% CI 0.777–0.954).

Table S3. Selected descriptive summaries of reviewer comments and recommended clarifications

Comment synthesis: Free-text comments were organized by the 12 question domains and summarized descriptively. One study investigator responsible for data analysis grouped comments expressing similar recommendations and drafted the summaries. A second study investigator verified the groupings, summary wording, and reported reviewer counts against the original written comments. Differences in interpretation were resolved by returning to the source comments; differing views and isolated safety concerns were retained. Theme frequencies are reported only for selected quantified themes. For each reported theme count, each reviewer was counted at most once, and only an explicitly expressed written recommendation or view contributed to the count. Silence and numerical appropriateness scores were not treated as endorsement. The denominator for reported theme counts was six reviewers. This descriptive synthesis did not require the reviewers to reach consensus or change their original ratings.

Interpretation: In Table 4, "Main recurrent reviewer theme" is a descriptive summary of written comments across all 12 domains. Where shown, theme counts such as 6/6 indicate the number of reviewers explicitly expressing the summarized recommendation or view. An omitted count means that a frequency is not reported; it implies neither zero endorsements nor unanimity. These counts are separate from the yes/no flag counts: a reviewer could recommend clearer wording without flagging the answer as potentially misleading or unsafe. Supplementary Table S3 provides expanded paraphrased summaries for six selected domains, with counts for selected additional themes; it is not an exhaustive list or a collection of verbatim quotations.

Domain	Summary of reviewer comments
Operative duration	All six reviewers considered 1–2 hours a reasonable typical range. The answer already stated that anatomy, surgical technique, and intraoperative difficulty could prolong the operation; reviewers recommended making that qualification more prominent and explicitly acknowledging other sources of complexity, including obesity, prior surgery, and medical instability.
Hospital stay	The answer already linked discharge timing to pain control, walking safety, medical condition, and support at home. All six reviewers regarded these qualifiers as appropriate but recommended foregrounding them so that the 1–3-day range would be less likely to be interpreted as an expected discharge date, particularly when medical stability or functional readiness is delayed.
Early mobilization	The answer already noted that weight-bearing and movement restrictions may alter mobilization. All six reviewers supported POD 0–1 mobilization as the usual target but recommended making medical and surgical causes of delay more explicit, including hypotension, anemia, nausea, neurologic status, or postoperative complications.
Assistive devices	Five of six reviewers supported a non-fixed, function-based approach to walker or cane use and recommended keeping discontinuation anchored to gait safety, muscle control, pain, and the absence of a significant limp.
Driving / return to work	The answer already noted the importance of laterality, analgesic use, braking ability, walking status, and job demands. Five of six reviewers recommended making the distinction between driving, desk work, and physically demanding work more explicit rather than implying a single return timeline.
Complications	Four reviewers suggested approximate risk ranges where defensible, while all agreed that individual risk should be contextualized by comorbidity and local practice.

Note: POD, postoperative day.
